# Perceptual inference, accuracy, and precision in temporal reproduction in schizophrenia

**DOI:** 10.1016/j.scog.2021.100229

**Published:** 2021-12-13

**Authors:** Natsuki Ueda, Kanji Tanaka, Kazushi Maruo, Neil Roach, Tomiki Sumiyoshi, Katsumi Watanabe, Takashi Hanakawa

**Affiliations:** a NCNP Brain Physiology and Pathology, Tokyo Medical and Dental University Graduate School, Yushima, Bunkyo-ku, Tokyo 113-8510, Japan; b Faculty of Arts and Science, Kyushu University, 744 Motooka, Nishi-ku, Fukuoka 819-0395, Japan; c Waseda Institute for Advanced Study, Waseda University, 1-21-1 Nishi Waseda, Shinjuku-ku, Tokyo 169-0051, Japan; d Faculty of Medicine, University of Tsukuba, 1-1-1 Tennodai, Tsukuba, Ibaraki 305-8577, Japan; e Faculty of Science and Engineering, Waseda University, 3-4-1 Okubo, Shinjuku-ku, Tokyo 169-8555, Japan; f Department of Preventive Intervention for Psychiatric Disorders, National Institute of Mental Health, National Center of Neurology and Psychiatry, 4-1-1 Ogawahigashi, Kodaira, Tokyo 187-8551, Japan; g Department of Advanced Neuroimaging, Integrative Brain Imaging Center, National Center of Neurology and Psychiatry, 4-1-1 Ogawahigashi, Kodaira, Tokyo 187-8551, Japan; h Department of Behavioral Medicine, National Institute of Mental Health, National Center of Neurology and Psychiatry, 4-1-1 Ogawahigashi, Kodaira, Tokyo 187-8551, Japan; i Faculty of Arts, Design, and Architecture, University of New South Wales, Cnr Oxford St & Greens Rd, Paddington, NSW 2021, Australia; j Integrated Neuroanatomy and Neuroimaging, Kyoto University Graduate School of Medicine, Yoshidakonoe-cho, Sakyo-ku, Kyoto 606-8501, Japan; k School of Psychology, University of Nottingham, NG7 2RD Nottingham, United Kingdom

**Keywords:** Central bias, Time perception, Schizophrenia, Uncertainty adjustment, Perceptual inference

## Abstract

Accumulating evidence suggests that deficits in perceptual inference account for symptoms of schizophrenia. One manifestation of perceptual inference is the central bias, i.e., the tendency to put emphasis on prior experiences over actual events in perceiving incoming sensory stimuli. Using an interval reproduction task, this study aimed to determine whether patients with schizophrenia show a stronger central bias than participants without schizophrenia. In the interval reproduction task, participants were shown a cross on a screen. The cross was replaced with a Gaussian patch for a predetermined time interval, and participants were required to reproduce the interval duration by pressing and releasing the space key. We manipulated the uncertainty of prior information using different interval distributions. We found no difference in the influence of prior information on interval reproduction between patients and controls. However, patients with SZ showed a stronger central bias than healthy participants in the intermediate interval range (approximately 450 ms to 900 ms). It is possible that the patients in SZ have non-uniform deficits associated with interval range or uncertainty of prior information in perceptual inference. Further, the severity of avolition and alogia was correlated with the strength of central bias in SZ. This study provides some insights into the mechanisms underlying the association between schizophrenic symptoms and perceptual inference.

## Highlights

• Schizophrenia produces stronger central bias in the intermediate interval range.
• Patients with schizophrenia exhibit low temporal precision.
• The strength of central bias negatively correlated with the severity of avolition.
• The strength of central bias positively correlated with the severity of alogia.
• Changes to activities of the prefrontal-striatal circuit may be responsible.

## 1 Introduction

Perceptual inference, defined as “*the ability to infer sensory stimuli from predictions that result from internal neural representations built through prior experience*” (Aggelopoulos, 2015), is considered a Bayesian process resulting from the overweighting of learned perceptual priors compared with incoming sensory stimuli, especially when the incoming sensory information is unreliable (Ashourian and Loewenstein, 2011; Schmack et al., 2013; Schmack et al., 2015). Perceptual inference may manifest as ‘central bias,’ which is the tendency for behavioral reports to gravitate towards the values of previous stimuli rather than the actual values of current stimuli (also called the central tendency effect). For example, in interval reproduction tasks, people with ideal mental health tend to overestimate shorter intervals but underestimate longer intervals, thereby shifting their reproduction times closer to the mean interval time within the experimental range (Cicchini et al., 2012; Jazayeri and Shadlen, 2010; Karaminis et al., 2016; Murai and Yotsumoto, 2018; Roach et al., 2017). Notably, perceptual inference may offer a new viewpoint for conceptualizing symptoms in schizophrenia (SZ) (Friston et al., 2016). It has been proposed that deficits of perceptual inference in the acquisition of perceptual expectation may lead to disruption of the internal model in SZ (Schmack et al., 2013; Schmack et al., 2015). However, previous investigations into perceptual inference in SZ identified no behavioral changes in patients with SZ when compared with healthy controls (HC) (Cassidy et al., 2018; Valton et al., 2019). Valton et al. (2019) demonstrated that the acquisition of perceptual priors by visual statistical learning is not disrupted in patients with SZ, and that the absence of behavioral changes related to perceptual inference may result from the effects of anti-psychotic medication. Cassidy et al. (2018) found that the severity of hallucinations in SZ correlated with perceptual bias; however, they were also unable to detect behavioral differences between HC and SZ relating to perceptual inference, possibly because the heterogeneous pathogenesis of SZ suppresses perceptual bias differences between HC and SZ.

Neuropsychological studies have identified distinct neural processes for millisecond- and second-range timing (Aso et al., 2010; Coull et al., 2011), wherein millisecond timing is associated with cerebellar circuits and supra-second timing is associated with basal ganglia circuits (Meck, 2005). Another dimension of time perception is that mechanisms of controlling the accuracy (deviation of estimates from the actual value) and precision (variability of reproduced duration) of timing may differ from each other (Coull et al., 2011). Notably, patients with SZ present with low accuracy and poor precision in time perception (Thoenes and Oberfeld, 2017), and the distortion of timing may span from ten milliseconds to several seconds (Ciullo et al., 2016). Therefore, we needed to account for these abnormalities in time perception as well as changes to the central bias when investigating perceptual inference in the time domain in patients with SZ.

In this study, we investigated how patients with SZ would infer and integrate prior and incoming temporal information in the ranges of millisecond and supra-second timing. We hypothesized that the strength of central bias in SZ may be incongruous with the change of distribution width because of deficits in the optimal integration of prior and sensory measurements. Using an interval reproduction task (Roach et al., 2017), we devised a method to quantitatively compare the strengths of central bias between a group of patients with SZ and a group of HCs. Our quantitative approach addressed the limitations of previous studies (Cicchini et al., 2012; Jazayeri and Shadlen, 2010), which predicated on Bayesian theories using only qualitative comparisons. Further, we aimed to determine the error characteristics of interval reproduction in SZ patients when compared with HCs as well as describe a correlation of central bias with the severity of SZ symptoms.

## 2 Methods

### 2.1 Participants

Eighteen patients with SZ (SZ group; age range: 19–49 years, diagnosed with ICD-10) and 43 adults without SZ (HC group; age range: 19–58 years) were enrolled in this study. All participants were naive to the study purpose and written informed consent was obtained from each participant according to the protocol approved by the Institutional Ethics Committee of the National Center of Neurology and Psychiatry. All participants completed the Japanese adult reading test (JART) to estimate premorbid intellectual ability (Table 1). In addition to JART, patients with SZ also completed neuropsychological and symptomatic assessments, including the Scale for the Assessment of Negative Symptoms (SANS), the Scale for the Assessment of Positive Symptoms (SAPS), and the Trail Making Test A and B (TMT-A and -B). The participants' and patients' demographic details are shown in Table 1.

**Table 1:** Demographic and clinical data of the patients and healthy controls.

Sociodemographic characteristics	Patients	Controls	Group comparison *p* Values
Sex (Female)	18 (12)	43 (22)	–
Age (mean ± SD)	31.94 ± 7.79	30.44 ± 13.83	0.463
JART (mean ± SD)	16.38 ± 4.73	20.37 ± 3.74	0.003
Dose of chlorpromazine equivalents^a^	643.29 ± 237.72	–	
Dose of amantadine equivalents	1.15 ± 2.52	–	
Outpatients/inpatients	2/17	–	
SAPS (mean ± SD)	19.27 ± 15.72	–	
SANS (mean ± SD)	25.94 ± 21.23	–	
TMT-A (mean ± SD; seconds)	43.44 ± 13.80	–	
TMT-B (mean ± SD; seconds)	75.16 ± 41.66	–	

a Five patients were being administered brexpiprazole, which has not been previously defined according to chlorpromazine-equivalent dosage. We estimated the chlorpromazine equivalents as follows: Wong et al. (2021) indicated that multiple doses of 2 mg/day and above of brexpiprazole are expected to result in dopamine D2/D3 receptor occupancies >80%. Multiple doses of 2 mg/day of brexpiprazole are equivalent to multiple doses of 18–24 mg/day of aripiprazole, which is expected to result in dopamine D2/D3 receptor occupancies of >80% (Gründer et al., 2008; Kegeles et al., 2008).

### 2.2 Experimental design

**Fig. 1:**
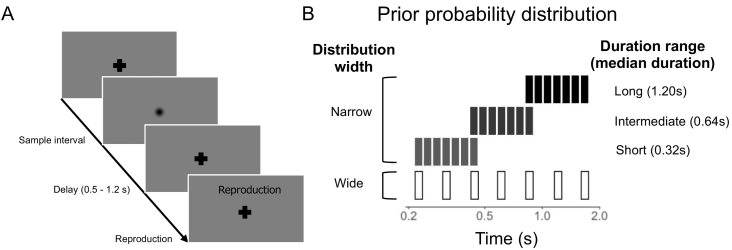
Experimental interval reproduction task. (A) Sequence of events during a trial. First, participants' attentions were fixated on a cross, and they were instructed to maintain fixation throughout the trial. After a random delay (0.5–1.2 s), a Gaussian patch (visual disk) was displayed for a specific interval (sample interval). Participants were instructed to reproduce the interval by pressing and holding the space key when the word ‘reproduction’ appeared. (B) Distribution of sample intervals. The wide distribution width was designed to produce high levels of uncertainty; the narrow distribution width was designed to produce low levels of uncertainty. The narrow distribution width comprised three interval ranges with different medians as follows: short interval range, 0.32 s; intermediate interval range, 0.64 s; and long interval range, 1.2 s. The wide distribution width comprised a single interval range with seven intervals having a median interval time of 0.64 s and a step size of log 0.15.

We employed an interval reproduction task (Roach et al., 2017) to investigate central bias over a range of time that included intervals with millisecond and supra-second timings. The task consisted of narrow and wide duration distributions (Fig. 1A) which introduced different levels of uncertainty to the priors.

In the HC group, we first determined the effects of prior distribution on perceptual reliability based on the strength of central bias, as demonstrated previously (Roach et al., 2017). Presumably, the strength of central bias is determined by the relative reliability of prior information and incoming sensory stimuli. To determine the effects of uncertainty of prior information on the strength of central bias, we fitted the data to a linear mixed model (LMM). Through these steps, we devised a method to quantitatively compare the strength of central bias between the SZ and HC groups. The estimation of the sample interval coefficient in the LMM is hereafter referred to as the “slope” and represents the strength of central bias. A smaller coefficient represents a near-horizontal slope and a stronger central bias. We sought to quantify the difference in slope between the HC and SZ groups. Specifically, we compared the degree of uncertainty adjustment represented by slope differences between two sample interval distribution widths to indicate participants' sensitivity to distribution width changes.

Patients with SZ are also reported to show impairments in time perception; therefore, we tested for error characteristics of interval reproduction. Reproduction errors ascribed to more general factors than perceptual inference were assessed using three indices (accuracy, precision, and total error), as described previously (Karaminis et al., 2016). Vertical error (VE), which refers to the degree of similarity between perceived and actual timing, was calculated as an accuracy parameter as previously described (Karaminis et al., 2016). As a precision parameter, coefficients of variation (CV) were computed for each distribution width. Additionally, we calculated the total reproduction error (TE), i.e., the combination of VE and CV. We hypothesized that the dissociation between actual and perceived timing (VE) would be larger than expected based on the strength of central bias. A participant's judgement of interval timing would rely more on prior knowledge for measurements with greater unreliability, representing a trade-off between VE and CV to reduce TE.

### 2.3 Stimuli and experimental procedures

Stimuli were displayed on an Apple MacBook Air. Visual stimuli comprised isotropic Gaussian patches (sigma = 10°) produced in MATLAB using the Psychtoolbox (Pelli, 1997) and displayed on gray backgrounds. Each participant viewed the display while seated approximately 60 cm away from the screen. Initially, the participant's attention was fixated on a cross in the display window. After a random delay (0.5–1.2 s), the cross was replaced with the isotropic Gaussian patch (disk) for a predetermined time interval. The participant was then required to reproduce the interval timing by holding down the space key. The time between pushing and releasing the space key was considered a behavioral measure of perceived duration (Fig. 1A).

The certainty of prior information is modulated by the width of prior interval distribution. The interval reproduction task consisted of narrow and wide interval distributions designed to introduce different levels of uncertainty to the priors. The two prior interval distribution widths were introduced as follows: one had a narrower distribution of sample intervals designed to produce lower levels of uncertainty, and the other had a wider distribution of sample intervals to produce higher levels of uncertainty. The narrow distribution width comprised three interval ranges with different medians as follows: short interval range, 0.32 s; intermediate interval range, 0.64 s; and long interval range, 1.2 s. In each interval range, seven intervals were sampled from a log-uniform distribution with a step-size of log 0.05 (i.e., log_10_ [median interval] ± 0.05). The wide distribution width comprised a single interval range with seven intervals having a median interval time of 0.64 s and a step size of log 0.15 (i.e., log_10_ 0.64 ± 0.15; Fig. 1B). Each of the seven sample intervals was presented 10 times in a pseudorandom order, and the order was semi-randomized among participants. In total, participants executed 280 trials (210 for the narrow distribution width and 70 for the wide distribution width). Participants were not informed of the duration distributions and were not given feedback during experimentation. Throughout the experiment, participants kept updating the priors and then adjusting the weighting on the priors or current stimuli based on the certainty level of incoming sensory measurements and of the priors (uncertainty adjustment).

### 2.4 Data analysis

#### 2.4.1 LMM analysis of the strength of central bias in HCs

For all statistical analyses, both the sample intervals and reproduced durations were logarithmically transformed to achieve Gaussian distributions. In line with a previous study by Karaminis et al. (2016), we used regression indices (i.e., slopes) as measures of the strengths of central bias. To this end, we computed an LMM wherein the reproduced durations were treated as outcome variables. Sample intervals, interval ranges, and interactions between sample intervals and interval ranges were considered as fixed effects. Participants' IQ levels estimated using JART (Ohi et al., 2017) were incorporated as fixed effects and covariates of no interest. The participants were the random effects. The covariance structure for repeated measurements was specified as compound symmetry. To account for model misspecification (e.g., heteroscedasticity), inferences were conducted using the robust sandwich variance method (Gosho and Maruo, 2018). Further, this method was used to control for unequal variance like during the measurement of intervals wherein the variance increased with increasing interval range. To evaluate the differences in slope across the interval ranges, we used a linear contrast vector of the interaction term between sample intervals and interval ranges. We then computed the ‘*uncertainty adjustment parameter*,’ which was defined as the difference in slope between the narrow and wide distribution widths.

#### 2.4.2 Accuracy and precision

Based on previous studies (Cicchini et al., 2012; Karaminis et al., 2016), we used the following three parameters to assess the error of reproduced durations: VE, which reflected a systematic offset from the sample interval in the vertical axis; CV, which reflected the scatter around the mean duration; and TE, which combined the effects of CV and VE.

For a given sample interval *i*, *VE*_*i*_ represents the difference between the sample interval and duration, which corresponds to the shift of the mean reproduced duration $\bar{R_{i}}\;$from a given sample interval *S*_*i*_, normalized by the average of sample intervals.

(1) $${\mathrm{VE}}_{i}=\frac{\left|\bar{R_{i}}-S_{i}\right|}{\bar{S}}.$$

Since the judgement of interval timing follows Weber's law (Haigh et al., 2021), we adopted CV in the analysis of precision. CV is given by the standard deviation of at each reproduced duration, normalized by the average of the sample interval.

(2) $$\begin{array}{c} {\mathrm{CV}}_{i}=\frac{\sqrt{\frac{\sum {\left(R_{i}-\bar{R_{i}}\right)}^{2}}{N}}}{\bar{S}}. \end{array}$$

The total root mean squared error, *TE*_*i*_, is given by the Pythagorean sum of VE and CV:

(3) $$\begin{array}{c} {\mathrm{TE}}_{i}=\sqrt{{\mathrm{VE}}_{i}^{2}+{\mathrm{CV}}_{i}^{2}}. \end{array}$$

#### 2.4.3 Between-group comparisons

We fitted the logarithmically transformed dataset to LMMs. Reproduced durations were treated as outcome variables; sample intervals, group designations, interval ranges, participants' IQ levels, interactions between sample intervals and interval ranges, interactions between interval ranges and group designations, and interactions between sample intervals, interval ranges, and group designations were treated as fixed effects. Participants were treated as random effects. We primarily investigated the differences in slope between the SZ and HC groups. In the slope analysis of prior distribution, we were especially interested in whether uncertainty adjustment parameters differed between the HC and SZ groups.

We then applied separate LMMs with VE, CV, or TE as outcome variables, respectively. The interval ranges, group designations, interactions between interval ranges and group designations, and participants' IQ levels were included as fixed effects; the participants were included as random effects.

#### 2.4.4 Analysis of correlation with severity of symptoms in SZ

We conducted a stepwise multiple regression to assess the relationship between the severity of SZ and the slope based on uncertainty adjustment. Each of the slopes, uncertainty adjustments, VEs, and CVs were included as outcome variables in different models, and SANS subscale scores, SAPS subscales, JART, TMT-A, and TMT-B were included as fixed effects; participants were included as random effects. Furthermore, we conducted an LMM analysis to consider the effects of behavior-modifying medications on behavioral indices. Each of the slopes and uncertainty adjustments were included as outcome variables in the different models, and the chlorpromazine-equivalent dosages were included as fixed effects; participants were included as random effects.

## 3 Results

### 3.1 Perceptual inference in HC

The effects of the sample interval, interval range, and the interaction between the sample interval and interval range were all significant in HC participants (*p* < 0.001 for all parameters; Table 3). The slope was significantly larger for the wide distribution width than the mean slope (mean of three interval ranges) for the narrow distribution width (*t* = 4.35; *p* *<* 0.001) in HC, indicating that HC performed uncertainty adjustment in accordance with prior distribution. The comparison of slopes across interval ranges within the narrow distribution width indicated that slope increased with decreasing interval lengths (long > intermediate > short), although the difference in slope between the long-and intermediate durations did not reach significance (intermediate-short: *t* = 1.98, *p* = 0.047; long-intermediate: *t* = 1.88, *p* = 0.061).

### 3.2 Comparison of perceptual inference between HC and SZ

Between-group comparisons of slope showed that the effects of sample intervals, interval ranges, group designations, and interval-by-interval range-by-group interactions were all significant (*p* < 0.05 for all parameters; Table 4). Multiple comparisons did not reveal a group-wise difference in the change in slope between the narrow and wide distribution widths (*t* = 0.55; *p* = 0.580), suggesting that uncertainty adjustment may not be altered in SZ. We further investigated group-wise differences in slope for each interval range. The SZ group returned smaller slope coefficients in the intermediate interval range (*t* = −2.67; *p* = 0.007), suggesting that the strength of central bias was greater in patients with SZ than in HCs. However, group-wise differences in the slope were nonsignificant for the short interval range (*t* = −0.63; *p* = 0.527), long interval range (*t* = −1.68; *p* = 0.093), and wide distribution width (*t* = −1.58; *p* = 0.114, corrected for multiple comparisons; Fig. 3).

### 3.3 Comparison of temporal accuracy and precision between HC and SZ

In our analysis of VE, there was no significant main effect of group designation, interval range, or group-by-interval range interaction (*p* > 0.05 for all parameters). However, the group-related effect on CV was significant (*p* < 0.001) while the effects of interval range and group-by-interval range interaction were nonsignificant (*p* *=* 0.105 and *p* = 0.394, respectively). The analysis of TE returned significant main effects of group designation (*p* *=* 0.003) and interval range (*p* < 0.487), and there was a group-by-interval range interaction (*t* = 0.002). Multiple comparisons in the narrow distribution width showed significant differences in TE between HC and SZ in the short interval ranges (*t* = −4.22; *p* = 0.001), but not in the intermediate interval ranges (*t* = −2.10; *p* > 0.999), long interval ranges (*t* = −1.03; *p* > 0.999), and wide distribution width (*t* = −3.03; *p* = 0.085; Fig. 4; Table 2 and Table 4).

**Table 2:** Mean and SD of CV, VE, and TE.

Mean ± SD in HC					Mean ± SD in SZ			
	Narrow-short	Narrow-intermediate	Narrow-long	Wide	Narrow-short	Narrow-intermediate	Narrow-long	Wide
CV	0.038 ± 0.012	0.028 ± 0.008	0.023 ± 0.006	0.038 ± 0.010	0.057 ± 0.025	0.044 ± 0.029	0.040 ± 0.033	0.060 ± 0.028
VE	0.045 ± 0.034	0.027 ± 0.017	0.020 ± 0.012	0.037 ± 0.017	0.077 ± 0.080	0.050 ± 0.058	0.026 ± 0.015	0.057 ± 0.023
TE	0.062 ± 0.031	0.041 ± 0.015	0.032 ± 0.010	0.054 ± 0.018	0.102 ± 0.075	0.073 ± 0.058	0.049 ± 0.034	0.084 ± 0.033

VE shows the vertical error, which refers to the dissociation between perceived timing and actual timing. CV shows the coefficients of variation which is a precision parameter. TE shows the total error which is the combined error of VE and CV.

**Table 3:** Results of the type III analysis of variance in the HC group to determine the effects of sample intervals, interval ranges, and interval-range interactions.

The results of type III analysis of variance table in HC								
	Slope		VE		CV		TE	
Value	F-Value	*P*-Value⁎, ⁎⁎	F-Value	P-Value	F-Value	P-Value	F-Value	P-Value
Sample interval	779.66	<0.001⁎⁎⁎	–	–	–	–	–	–
Interval ×Interval range	8.81	<0.001⁎⁎⁎	–	–	–	–	–	–
Interval range	7.33	<0.001⁎⁎⁎	28.11	<0.001⁎⁎⁎	43.07	<0.001⁎⁎⁎	44.29	<0.001⁎⁎⁎
IQ	0.07	0.787	0.61	0.437	0.15	0.693	0.55	0.460

HC, healthy controls group; VE, vertical error; CV, coefficients of variance; TE, total error.

⁎ p < 0.05.

⁎⁎ *p* < 0.01.

⁎⁎⁎ *p* < 0.001.

**Table 4:** Results of the type III analysis of variance between groups to assess the effects of sample intervals, interval ranges, group designations, and interval-by-interval range-by-group interactions on the strength of central bias measured by the slope of the linear mixed model.

The results of type III analysis of variance including between group (HC and SZ) analysis								
	Slope		VE		CV		TE	
Value	F-Value	P-Value	F-Value	P-Value	F-Value	P-Value	F-Value	P-Value
Sample interval	311.48	<0.001⁎⁎⁎	–	–	–	–	–	–
group	5.16	0.026⁎	3.75	0.057	11.13	0.001⁎⁎	9.23	0.003⁎⁎
Interval range	10.96	<0.001⁎⁎⁎	0.54	0.655	1.00	0.394	0.81	0.487
IQ	0.10	0.758	2.64	0.109	4.51	0.037⁎	4.11	0.047⁎
Group × Interval range	2.99	0.032⁎	2.60	0.053	2.07	0.105	5.11	0.002⁎⁎
Interval × Interval range	12.55	<0.001⁎⁎⁎	–	–	–	–	–	–
Interval × Group	4.01	0.045⁎	–	–	–	–	–	–
Interval × Interval range × group	2.76	0.040⁎	–	–	–	–	–	–

VE, vertical error; CV, coefficients of variance; TE, total error.

⁎ p < 0.05.

⁎⁎ p < 0.01.

⁎⁎⁎ p < 0.001.

**Fig. 2:**
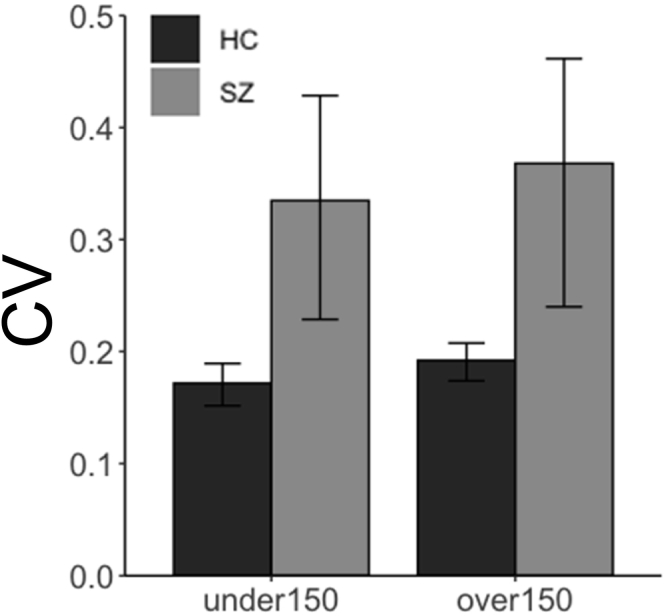
Mean coefficient of variance (CV) for trial N and N-1. Error bars show bootstrapped 95% confidence intervals. HC, healthy controls group; SZ, patients with schizophrenia group.

The time differences between intervals of successive trials could be as short as 50 or 100 ms. It was possible that patients with SZ may find difficulty in distinguishing such small timing differences because of condition-dependent deficits in detecting temporal asynchronies and judging temporal order. In these cases, patients with SZ may repeat the response duration and the CV would be small. Therefore, we conducted a sub-analysis to identify changes in CV when the interval timing between successive trials N and N-1 differed by more or less than 150 ms. We generated an LMM where the CV was treated as the outcome variable; the fixed effects were interval timing differences between successive trials (over 150 ms vs under 150 ms), group designations, participants' IQ levels, and interactions between interval timing differences and group designations, and participants were included as the random effect. We found significant effect of the group designation (*F* = 6.46, *p* = 0.012) but interval timing differences, IQ, and the interaction between interval timing differences and group designations was not significant on CV (*p* > 0.05; Fig. 2). Previous studies showed that patients' temporal discrimination abilities for visual stimuli are better than for auditory stimuli; the 75% threshold was 140 ms for auditory stimuli while the 75% threshold was 45 ms for visual stimuli (Stevenson et al., 2017). Therefore, the effect of response time repetition in patients with SZ was negligible in our study design.

**Fig. 3:**
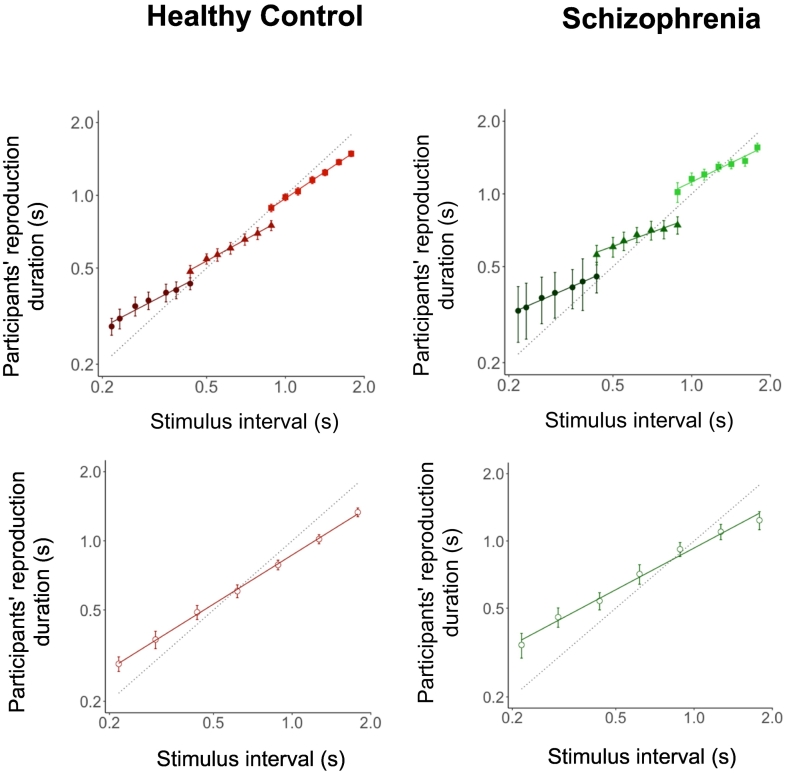
Mean reproduction durations of sample intervals in the narrow and wide distribution widths wherein the slope of the line provides an index for the strength of central bias. Error bars show bootstrapped 95% confidence intervals. Solid lines show best-fitting linear regressions for each range, whereas the dotted diagonal lines denote veridical (unbiased) performance. The filled circles show the values for the short interval range, the triangles show values for the intermediate interval range, the squares show values for the long interval range, and the open circles show values in the wide distribution width. HC, healthy controls group; SZ, patients with schizophrenia group. HC, healthy controls group; SZ, patients with schizophrenia group.

**Fig. 4:**
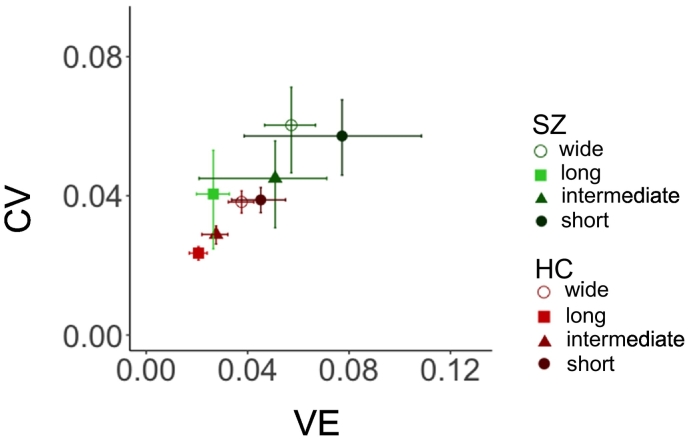
CV versus VE for the reproduced durations of intervals from the narrow and wide distribution widths. VE (vertical error) refers to the degree of similarity between perceived and actual timing, and CV (coefficients of variation) indicates the precision of perceived timing. Error bars show bootstrapped 95% confidence intervals. The filled circles show the values for the short interval range, the triangles show values for the intermediate interval range, the squares show values for the long interval range, and the open circles show values in the wide distribution width. HC, healthy controls group; SZ, patients with schizophrenia group; CV, coefficients of variance; VE, vertical error.

### 3.4 Correlation analysis

We conducted a stepwise multiple regression analysis to investigate the correlation of SZ symptom severity with the strength of central bias and uncertainty adjustment. There was no significant difference in uncertainty adjustment between the SZ and HC groups, probably because of the heterogeneity of SZ symptoms (Edwards et al., 2016). The slope value correlated with alogia (*t* = −2.86; *p* = 0.012) and avolition (*t* = 2.85; *p* = 0.012), but there was no correlation between symptom severity and uncertainty adjustment (*p* > 0.05 for all symptoms tested). There were also no correlations between the chlorpromazine-equivalent dosages and all behavioral indices (*p* > 0.05 for all indices).

## 4 Discussion

Using the interval reproduction task, we demonstrated that patients with SZ showed stronger central bias than HCs in the intermediate interval range only. Overall, patients with SZ demonstrated low temporal precision. Correlation analysis showed that negative symptoms were associated with the strength of central bias in SZ.

### 4.1 Perceptual inference influenced by time range in HCs

The analysis of LMM slopes in the HC group found that interval ranges affected the strength of central bias within the narrow distribution width. The slopes became steeper as sample intervals shifted from the short to long interval ranges, suggesting a lessened strength of central bias in the long interval range than in the short interval range. This result is inconsistent with that of a previous study (Jazayeri and Shadlen, 2010), which indicated that longer sample intervals were associated with stronger central bias. The discrepancy may result from differences in the distribution of sample intervals; Jazayeri and Shadlen (2010) used arithmetic sequences while we adopted logarithmically spaced sequences. Duration distributions based on arithmetic sequences gradually become more difficult to discriminate in longer interval ranges, which may interrupt temporal precision (Jazayeri and Shadlen, 2010).

### 4.2 Between-group perceptual inference in relation to temporal errors

There were no significant between-group differences in the uncertainty adjustment parameters by LMM slope analysis, which is consistent with the findings of a previous study that used a probabilistic inference task (Valton et al., 2019). The finding failed to support the idea that patients with SZ may have statistical deficits in perceptual inference induced by ambiguous changes of external stimuli. According to the analysis of slope differences across interval ranges, the increased strength of central bias in the SZ group compared with that of the HC group was limited to the intermediate interval range (approximately 450 ms to 900 ms). In the short interval range (approximately 220 ms to 450 ms), we found that the TE in the SZ group was significantly higher than in the HC group. The abnormally elevated reproduction errors appeared to interfere with accurate measurements of central bias in the SZ group. We found that patients with SZ returned higher CVs compared with those of HCs, while between-group differences in the strength of central bias in the long interval range (approximately 1000 ms to 1800 ms) and wide distribution width (approximately 220 ms to 1800 ms) were nonsignificant. Previous studies have shown that the strength of central bias decreased and temporal discrimination in patients with SZ improved with age and developing expertise (Cicchini et al., 2012; Karaminis et al., 2016). Our findings seem to indicate a lower sensitivity of perceptual variability in certain interval ranges (or certain distribution widths) or a fragility of temporal expectation in patients with SZ. Previous studies support the notion of fragility of temporal expectation (Martin et al., 2017) and this heterogeneity of perceptual inference in SZ should be considered in future studies.

It remains unclear whether the weighting of perceptual priors and incoming sensory stimuli based on perceptual precision in perceptual inference reflects the patients' subjective evaluations of performance precision. Further study is needed to investigate the difference between objective and subjective evaluations of performance precision in patients with SZ.

### 4.3 Relationship between schizophrenic symptoms and strength of the central bias

Studies have shown that the disturbance of perceptual inference in SZ adds a new dimension to explain patient cognition and SZ symptoms (Powers et al., 2017; Schmack et al., 2013; Schmack et al., 2015; Valton et al., 2019). Although we did not find convincing group-wise differences in uncertainty adjustments or a correlation with SZ symptoms, we did find a correlation between slope and SZ symptom severity, specifically the severity of avolition and alogia. The slope was positively correlated with avolition, indicating that the effects of central bias are smaller in cases of greater avolition severity. Avolition is a lack of motivation associated with reward anticipation. Avolition severity has been found to negatively correlate with ventral striatal activation (Simon et al., 2010), and striatal dopamine release is related to perceptual priors in perceptual inference in time perception (Cassidy et al., 2018). These findings suggest that the association between reduced central bias and severe avolition may be mediated by reduced ventral striatal activity, which is associated with perceptual inference and reward anticipation (Apaydin et al., 2018; Ting et al., 2015).

Moreover, the slope was inversely correlated with alogia, indicating that the effects of central bias increased with increasing severity. Alogia is an index of speech poverty. The severity of alogia is associated with hyperactivity of the medial frontal areas (Liemburg et al., 2015), and the medial frontal cortex is associated with perceptual prior and prediction errors (Zuanazzi and Noppeney, 2019). Hence, the strength of central bias in SZ may be associated with hyperactivity of the medial frontal cortex, which may be related to altered perceptual priors. The prefrontal-striatal circuit, including the medial prefrontal cortex and dorsal striatum, integrates the reward and timing systems of the brain (Apaydin et al., 2018). Our results suggest that both avolition and alogia are likely related to the strength of perceptual priors, and these correlations may be the result of alterations in the activities of the prefrontal-striatal circuit. Further research is needed to clarify the relationship between alterations in the prefrontal-striatal circuit, perceptual inference, and negative SZ symptoms.

### 4.4 Study limitations

A potential limitation of our study is the effect of antipsychotic medications on time perception and behavioral dynamics. Although we found no correlations between medications and behavioral indices, the effects of antipsychotic medication are thought to be mediated by antagonist activity against the dopamine D_2_ receptor in the striatum, which plays an important role in motor control and time perception (Inagawa et al., 2020; Peluso et al., 2012). Therefore, we cannot exclude the possibility that antipsychotic medications altered the timing performances of patients with SZ.

## 5 Conclusions

The identification of increasing strengths of central bias were limited in patients with SZ. Patients with SZ appear to have heterogeneous deficits associated with different interval ranges or in the uncertainty of prior information in perceptual inference. Notably, we identified a correlation between the strength of central bias and SZ symptom severity. These findings may provide a novel view of the relationship between SZ symptoms and neural mechanisms underlying temporal processing.

## Funding

This work was supported by 10.13039/501100001691JSPS KAKENHI (Grant Number: JP18K13363) to U.N. and 20H03610 to T.S. from the 10.13039/501100001700Ministry of Education, Culture, Sports, Science, and Technology, Japan, and AMED Brain/MINDS Beyond (21 dm0307003h0004) to H.T.

## CRediT authorship contribution statement

N.U., K.T., and T.H. conceived and planned the experiments. N.U., and K.T.

carried out the experiments. K.M. and N.U. performed the analytic calculations.

N.R., K.T., T.S., and K.W. helped supervise the project. T.H. supervised the project.

## Declaration of competing interest

The authors declare no conflict of interest.
